# Obituary

**Published:** 1863-08

**Authors:** 


### OBITUARY.

Killed by a gun shot on the 18th of July 1863, at Wythesville, Western Virginia, Col. Jno. T. Toland, formerly of this city.

Col. T. was for a number of years engaged in the Dental furnishing business, and was well known to the dental profession throughout the country, and especially in the West. Of his virtues it is needless to speak here; all who knew him, know what he was for frankness, was a prominent trait in his character.

He entered the army shortly after the outbreak of the rebellion, and was in constant service from that till the time of his death. He was a brave and valiant officer; he was always foremost when danger was thickest, and pressed where duty seemed to call irrespective of personal safety.

In his death the Dental profession lost a friend, and one who was ever on the alert to guard, protect and further its interests. Society lost a warm-hearted, social and genial member, and the army a brave and noble officer; whose place will not be easily filled. It is hard to realize that he is gone. T.

